# Leaf‐Inspired Flexible Thermoelectric Generators with High Temperature Difference Utilization Ratio and Output Power in Ambient Air

**DOI:** 10.1002/advs.202004947

**Published:** 2021-05-09

**Authors:** Qing Zhou, Kang Zhu, Jun Li, Qikai Li, Biao Deng, Pengxiang Zhang, Qi Wang, Chuanfei Guo, Weichao Wang, Weishu Liu

**Affiliations:** ^1^ Department of Materials Science and Engineering Southern University of Science and Technology Shenzhen 518055 China; ^2^ Key Laboratory of Energy Conversion and Storage Technologies (Ministry of Education) Southern University of Science and Technology Shenzhen 518055 China; ^3^ Department of Electronics and Tianjin Key Laboratory of Photo‐Electronic Thin Film Device and Technology Nankai University Tianjin 300071 China

**Keywords:** flexible thermoelectric generators, heat transfer, human body power, temperature difference utilization ratio, wearable electronics

## Abstract

The inherently small temperature difference in air environment restricts the applications of thermoelectric generation in the field of Internet of Things and wearable electronics. Here, a leaf‐inspired flexible thermoelectric generator (leaf‐TEG) that makes maximum use of temperature difference by vertically aligning poly(3,4‐ethylenedioxythiophene) polystyrene sulfonate and constantan thin films is demonstrated. Analytical formulae of the performance scales, i.e., temperature difference utilization ratio (*φ*_th_) and maximum output power (*P*_max_), are derived to optimize the leaf‐TEG dimensions. In an air duct (substrate: 36 °C, air: 6 °C, air flowing: 1 m s^−1^), the 10‐leaf‐TEG shows a *φ*_th_ of 73% and *P*_max_ of 0.38 µW per leaf. A proof‐of‐concept wearable 100‐leaf‐TEG (60 cm^2^) generates 11 µW on an arm at room temperature. Furthermore, the leaf‐TEG is flexible and durable that is confirmed by bending and brushing over 1000 times. The proposed leaf‐TEG is very appropriate for air convection scenarios with limited temperature differences.

## Graphical abstract

A leaf‐structure thin film‐based flexible thermoelectric generator (leaf‐TEG), structure similar to fins, enhances the temperature difference utilization ratio (*φ*_th_) of up to 85% with a rapid response to air temperature fluctuations. Thus opening the path to the development of highly efficient output under limited temperature difference conditions for the flexible TEG field.

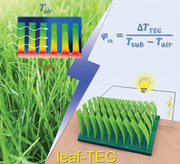

## 1 Introduction

Thermoelectric power generation (TEG) provides convenient electricity by directly utilizing the temperature difference from the environment that could be used for the independent operation of internet of things (IoT) sensors and wearable electronics.^[^ ^1^, ^2^, ^3^, ^4^, ^5^, ^6^ ^]^ In the wearable scenario, flexibility is essential for TEG devices. Various flexible TEGs have been previously reported, including bulk thermoelectric (TE) legs plus flexible electrodes, fiber‐based devices, and thin film‐based devices. Among them, bulk Bi_2_Te_3_‐based TE legs with flexible electrodes have exhibited the highest output power,^[^ ^7^, ^8^, ^9^ ^]^ but has a limited comfortable capability. Eom et al.^[^ ^7^ ^]^ reported a bracelet‐like module of a flexible TE system with a copper heat sink that generated 3.2 µW cm^−2^ when worn on the wrist at a slow running pace. However, the inclusion of a heat sink adds additional weight and space. Suarez et al.^[^ ^9^ ^]^ used a liquid metal to interconnect the devices with an aim of increasing flexibility, to which the devices were fastened to the wrist and produced maximum voltages and powers of 1.47 mV and 0.37 µW cm^−2^ at an ambient temperature of 24 °C, respectively. However, the maximum temperature difference across the TE legs (∆*T*_TEG_) was only 0.4 °C, and the temperature difference utilization ratio *φ*_th_ which is defined as the ratio of the ∆*T*_TEG_ and the available temperature difference (∆*T*) between the heat sink and heat reservoir, i.e., *φ*_th_ = ∆*T*_TEG_/∆*T*, was only 5%. Although the fiber‐based flexible TEG provides promising applicability in increasing the flexibility of the fiber‐woven TEG, such as with real cloth.^[^ ^10^, ^11^, ^12^, ^13^ ^]^ However, the output power is low as compared with bulk TEG. Sun et al.^[^ ^14^ ^]^ reported stretchable fibers woven TEG by using poly(3,4‐ethylenedioxythiophene) polystyrene sulfonate (PEDOT:PSS) and oleamine‐doped carbon nanotube fibers, which showed a peak power density of 7 µW cm^−2^ for a large ∆*T*_TEG_ of 44 °C. Moreover, these reported power density data were based on the contact heat transfer mode with ideal constant temperature boundary conditions, rather than the actual wearable service environment.

Thin film‐based flexible TEGs present a balance between TE performance and mechanical flexibility. Currently, most flexible thin film based TEGs have in‐plane architecture with all the TE legs and metal interconnections lying on a substrate to obtain the temperature difference.^[^ ^15^, ^16^, ^17^ ^]^ In general, researchers use a cold sink and a heat source to create contact between the two ends of the TEGs across a plane. However, it is difficult to obtain ∆*T* through this in‐plane architecture due to its parallel arrangement to the skin surface in the case of wearing, which limits the *φ*_th_. In an indoor environment, the ∆*T* between human skin and air is usually about 10 °C, whereas temperature difference on TE materials(∆*T*_TE_) is only ≈1 °C for conventional bulk devices with a cooling fin,^[^ ^18^ ^]^ which corresponds to an *φ*_th_ of ≈10%. Therefore, it is critical to increase temperature difference on TE materials through a system level design by considering heat exchange among the TEG device, heat source, and heat sink. There are few reports of vertically assembly film‐based TEGs.^[^ ^19^, ^20^, ^21^, ^22^, ^23^ ^]^ Fang et al.^[^ ^24^ ^]^ designed Rolled modules is easy to establish temperature difference, but both two sides of the device cannot directly exchange heat with air and sacrificed most of the flexibility. Ren et al.^[^ ^25^ ^]^ recently reported a Lego‐like wearable thermoelectric generator which can directly exchange heat with air, while they did not discuss the thermal management constraints between device geometry and the environmental conditions.

In this article, we proposed flexible leaf‐TEG that could vertically stand on a substrate by using flexible p‐type organic PEDOT:PSS film and n‐type inorganic constantan alloy thin film, which was inspired from grass leaves. This leaf‐TEG structure holds the advantage of cooling fin that could make maximum use of the temperature difference in the actual environment. We proposed a performance scale, i.e., temperature difference utilization ratio *φ*_th_, for the leaf‐TEG. We theoretically investigated the connections between *φ*_th_ and the synergistic effect of TE legs (TE‐leaf) length (*L*) and working environment, such as *T*_air_ and *v*_air_. Experimentally, we designed an air duct with tunable air flowing temperature and velocity for power generation test. The measured *φ*_th_ and maximum output power (*P*_max_) showed a good consistence with theoretical predication. Furthermore, over 1000 bending and brushing tests was carried out to confirm the flexibility and durability. Finally, the power generations in the palm touching, mouth blowing, and arm wearing were conducted, suggesting that the proposed leaf‐TEG is good for energy harvesting from ambient air environment.

## 2 Results

### 2.1 Design of the Leaf‐TEG

**Figure 1:**
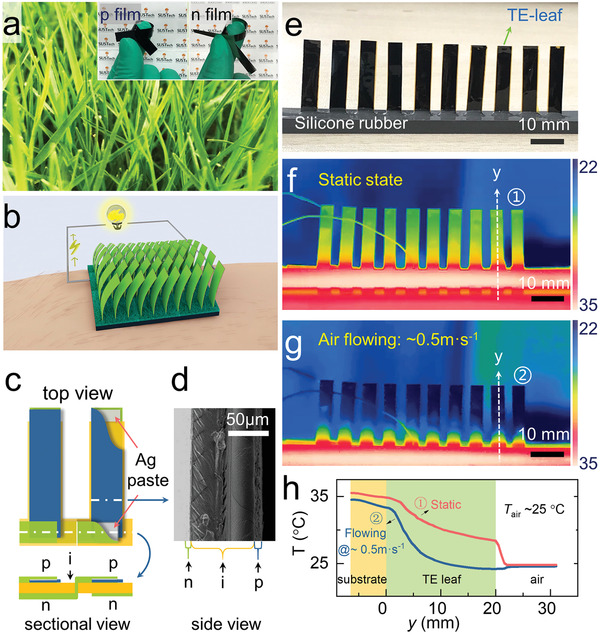
Structure and the temperature distribution of leaf‐TEG. a) Grass leaves and TE flexible films. b) Concept of leaf‐TEG working under wearing condition. c) The detailed structure schematic of proposed leaf‐TEG consisting of “p” and “n” TE films separated by double‐sided‐polyimide‐tape “i”. The partial section view shows circuit connections. d) Scanning electron microscope photograph of the TE‐leaf in side view. Pores (dark areas in p‐film) in ionic liquids modified PEDOT:PSS free‐standing film improves the bending deformation ability of the film. e) Leaf‐TEG assembling with 10 back‐to‐back TE couples. f,g) Infrared photograph of leaf‐TEG under a static state and flowing air of 0.5 m s^−1^ at room temperature (≈25 °C) placed on a hotplate (36 °C). h) The temperature distribution of leaf‐TEG oriented the heat flow, as *y*‐axis shown in f) and g).

Maximizing the utilization of temperature difference serves as a prerequisite for energy harvesting from environmental by using TEGs. Therefore, it is of significance to search for TEG structures that can efficiently capture temperature differences from the ambient environment. The flexible leaves of gramineous plants (**Figure** **1a**) can effectively generate and maintain an appreciable temperature difference in the natural environment. As an example, leaf tips are ideal sites for the condensation of dewdrops,^[^ ^26^, ^27^ ^]^ since they can closely follow the temperature drop of the air in the early morning with the help of the slender shapes and efficient surface heat exchange, while the bottom temperature is kept close to the ground. Inspired by the grass leaves, we proposed a leaf‐TEG (Figure 1b), which vertically stands on the heat source and exhibits both high flexibility and superior capability of establishing a large temperature difference. The detailed structure schematic of proposed leaf‐TEG is as show in Figure 1c. Here we use a p‐type PEDOT:PSS free‐standing film(“p” in Figure 1c),^[^ ^28^, ^29^ ^]^ and an n‐type constantan film (“n” in Figure 1c), and an electrically insulating layer (90 µm thick double‐sided‐polyimide‐tape, marked as “i” in Figure 1c) to make leaf‐TEG. The fabrication process of the large‐area flexible PEDOT:PSS film and thermoelectric properties of PEDOT:PSS and constantan thin films are shown in Figures S1 and S2 (Supporting Information), respectively. The assembling process is shown in Figure S3 (Supporting Information). The structure and thickness of TE‐leaf was confirmed by the cross‐sectional scanning electron microscope photograph (Figure 1d). The organic–inorganic hybrid structure balances the thermoelectric performance and mechanical strength. To achieve stable thermal contact with heat sources, the TE leaves were planted in a flexible silicone rubber substrate with the thermal conductivity of ≈2 W m^−1^ K^−1^ (Figure 1e).

Figure 1f–h shows the temperature profile of a 10‐leaf‐TEG (with 10 identical TE leaves, *L* = 20 mm) with 10 TE leaves serially connected, which was taken by a thermal imaging camera. We set the substrate temperature at 36 °C close to human skin temperature in an indoor environment (25 °C). The detailed experiment setup is shown in Figure S4 (Supporting Information). The temperature difference across the TE‐leaf is about 6 °C in the static state, corresponding to a *φ*_th_ of 58%. When the air velocity *v*_air_ is 0.5 m s^−1^, *φ*_th_ increases significantly to 85%. For comparison, the measured *φ*_th_ of a commercial TEG module is only 8% at the same static condition, and 9% for *v*_air_ of 0.5 m s^−1^ (Figure S5 and Table S1, Supporting Information). It clearly suggests the superiority of the leaf‐TEG in effectively utilizing the temperature difference in the ambient atmosphere, which takes the advantage of conventional cooling fins while maintaining its flexibility.

### 2.2 Power Generation Performance and Corresponding Temperature Difference Utilization Ratio

**Figure 2:**
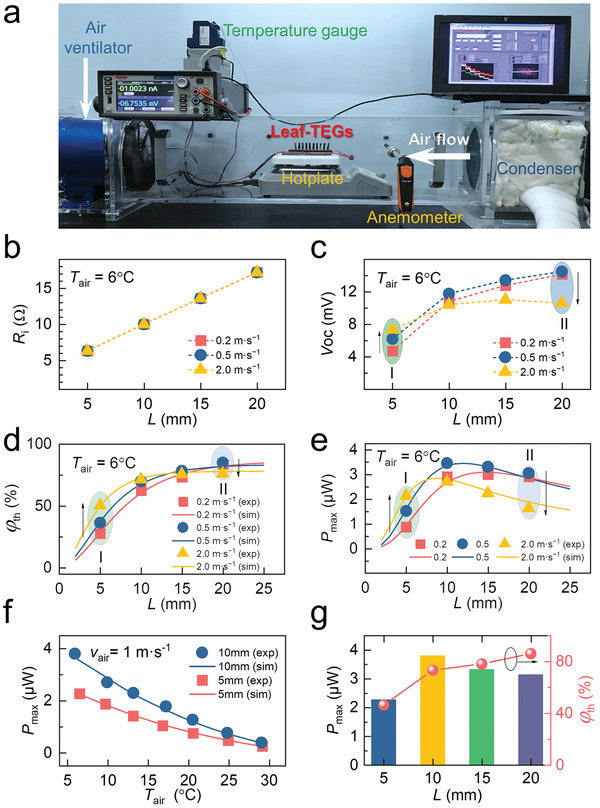
Effects of *v*_air_ and *T*_air_ on the output power performance of leaf‐TEG with different dimensions. a) A lab‐made air duct system that can be used to adjust *T*_air_ and *v*_air_. From b) to e), *T*_air_ is 6 °C. b) Internal resistance *R*_i_, c) Open‐circuit voltage *V*_oc_, d) temperature difference utilization ratio *φ*_th_, and e) maximum output power *P*_max_ of leaf‐TEG with different length under different air velocity conditions (0.2, 0.5, and 2.0 m s^−1^). f) Maximum output power varies with the air temperature. g) The maximum *P*_max_ and the corresponding *φ*_th_ of leaf‐TEG (*L* = 5, 10, 15, and 20 mm) in our testing conditions (*T*_air_: 6–29 °C, *V*_air_: 0.15–2.0 m s^−1^).

**Figure** **2a** shows a lab‐made air duct system that was used to simulate different environmental conditions with controllable air velocity and air temperature. Four 10‐leaf‐TEG devices with different TE‐leaf length (5, 10, 15, and 20 mm) were measured under different air condition (*v*_air_: 0.15, 0.2, 0.25, 0.3, 0.35, 0.4, 0.45, 0.5, 1.0, 1.5, and 2.0 m s^−1^, *T*_air_: 6, 9, 13, 17, 21, 25, and 29 °C). Each TE‐leaf has the same thickness of 0.15 mm (PI tape: 0.095 mm, N film: 5 µm and P film: 50 µm) and the same width of 4 mm. All the measured data are given in Figure S6 (Supporting Information). Here, we select a set of representative data measured under *T*_air_ of 6 °C to make a clear comparison as shown in the scattered data in Figure 2b–e.

Figure 2b shows the linearly increased internal resistance (*R*_i_) of the10‐leaf‐TEG from 6.3 to 10.1, 13.6, and 17.2 Ω as the TE‐leaf length of the device increases from 5 to 10, 15, and 20 mm, respectively. In the as‐fabricated leaf‐TEG, internal resistance comprises of the electric resistance of p–n couples and extra resistance (*R*_e_) due to the contact resistance and metal interconnector (i.e., silver paste in this study), i.e., *R*_i_ = *N* × (1/*σ*_p_ × *L_p_*/*A*_p_ + 1/*σ*_n_ × *L*_n_/*A*_n_) + *R*_e_, where *A*, *L*, *N*, and *σ* are the cross sectional area, TE‐leaf length and leaf number, electrical conductivity, respectively. The estimated extra resistances of 10‐leaf‐TEG are around 2.3 Ω for all the four devices, suggesting a repeatable devices assembling process. The as‐fabricated leaf‐TEG is also very robust with negligible fluctuation of the internal resistance during the measurement under different air velocities (0.2–2 m s^−1^).

Figure 2c shows the open circuit voltage (*V*_oc_) of the 10‐leaf‐TEG with different TE‐leaf lengths. Under an air flowing condition (*v*_air_ = 0.2 m s^−1^, *T*_air_ = 6 °C), the *V*_oc_ significantly increases from 4.7 to 10.8 mV as the *L* increases from 5 to 10 mm, and then shows a saturated tendency with a value of 14.2 mV for the one with *L* of 20 mm. However, the relationship between the *V*_oc_ and *v*_air_ is more complicated. The increase of *v*_air_ from 0.2 to 2.0 m s^−1^ increases the *V*_oc_ for the 10‐leaf‐TEG with a short leaf of 5 mm (I in Figure 2c), while decrease the *V*_oc_ for the one with longer leaf of 20 mm (II in Figure 2c). Since the *V*_oc_ is directly connected with effective temperature across the leaf, i.e. ∆*T*_TE_, through the relation of *V*_oc_ = *N*∙(*S*_p_ *+S*_n_)∙∆*T*_TE_. Increasing *v*_air_ has positive effect on air convective heat transfer coefficient and hence lager ∆*T*_TE_. On the other hand, increasing *v*_air_ could cool the substrate and reduce the ∆*T*_TE_. Such a cooling effect is also observed in our skin.^[^ ^30^ ^]^ Both the convective heat transfer and substrate cooling effect have some connection with the TE‐leaf length, resulting in the complicated relationship among the *V*_oc_, *v*_air_, and *L*. Figure 2d shows the *φ*_th_ of 10‐leaf‐TEG with different TE‐leaf lengths, which is estimated from the relation *φ*_th_ = ∆*T*_TE_/∆*T* = *V*_oc_/*N*/(*S*_p_ *+S*_n_)/(*T*_sub_‐*T*_air_). Similar to the *V*_oc_, the *φ*_th_ shows a significant increase as the *L* increased from 5 to 10 mm, and then a saturated tendency. The 10‐leaf‐TEG with *L* = 20 mm shows a highest *φ*_th_ ranged from 76% to 86% depending on the *v*_air_ as *T*_air_ = 6 °C. Figure 2e shows the optimized output power *P*_max_ (i.e.*, P*_max_ = *V*_oc_^2^/4*R*_i_ = [(*S*_p_ *+S*_n_)*∙*(*T*_sub_‐*T*_air_)]^2^/4 × *φ*_th_^2^/*R*_i_) of 10‐leaf‐TEG with different TE‐leaf lengths. It is noted that both the *φ*_th_ and *R*_i_ significantly depend on the TE‐leaf length. Generally, leaf‐TEG with shorter leaves has smaller *φ*_th_ and *R*_i_, while the one with longer leaves has larger *φ*_th_ and *R*_i_. Under a nature air convention flowing condition (*v*_air_ = 0.2 m s^−1^, *T*_air_ = 6 °C and *T*_sub_ = 36 °C), the *P*_max_ changed from 0.9 to 2.9, 3.0, and 2.9 µW as *L* increased from 5 to 10, 15, and 20 mm, respectively. While, for the forced air convention flowing condition (*v*_air_ = 2 m s^−1^, *T*_air_ = 6 °C and *T*_sub_ = 36 °C), the *P*_max_ changed from 2.1 to 2.7, 2.2, and 1.6 µW as *L* increased from 5 to 10, 15, and 20 mm, respectively.

It is clearly suggested that the dimension choice of the leaf strongly depends on the service environment. Comparing to air velocity, the influence of air temperature on output power is relatively simple as shown in Figure S7 (Supporting Information). When the air temperature became 25 °C, the *v*_air_ and TE‐leaf length affected the output power performance in a similar way of *T*_air_ = 6 °C, as shown Figure S8 (Supporting Information). Figure 2f shows air temperature dependent output power of the 10‐leaf‐TEG with two TE‐leaf length of 5 and 10 mm at a given *v*_air_ = 1.0 m s^−1^. Among the investigated conditions of *v*_air_ and *L*, the highest output power of 10‐leaf‐TEG is 3.8 µW as *v*_air_ = 1 m s^−1^ and *L* = 10 mm with fixed *T*_air_ = 6 °C and *T*_sub_ = 36 °C, which corresponds to *P*_max_ = 0.38 µW for a single leaf and *φ*_th_ = 73% (see in Figure 2g).

### 2.3 Heat Transfer Model and Theoretical Analysis

To get a comprehensive knowledge of the operating characteristics of the leaf‐TEG, here we used the 1D fin heat transfer theory to investigate temperature difference within a leaf‐TEG standing in a static or flowing air environment, and derived the explicit formulae of the *φ*_th_ and the maximum output power *P*_max_. This 1D model fits well with Zhu's general design strategy for matching the external thermal resistance, output power and voltage of TEGs on system level.^[^ ^31^ ^]^ As shown in Figure S9 (Supporting Information), the TE‐leaf absorbs heat from the substrate and continuously releases heat at its lateral surface, resulting in a descending temperature profile. By solving the steady‐state governing equation and boundary conditions (Note S3 in the Supporting Information), the temperature distribution along the length of the TE‐leaf was obtained

(1) $$T=\frac{h_{b}}{h_{b}+\kappa_{\mathrm{eff}}\beta \tanh\left(\beta L\right)}\frac{\cosh\left(\beta y-\beta L\right)}{\cosh\left(\beta L\right)}\Delta T+T_{\;\mathrm{air}}$$

Where *h*_b_ is the equivalent heat transfer coefficient between the TE‐leaf root and the heat source; *κ*_eff_ is the effective thermal conductivity of the TE‐leaf, which could be calculated by a weighted average of the thermal conductivities of the three components; *βL* is a dimensionless parameter with *L* being the TE‐leaf length and $\beta =\sqrt{h_{\mathrm{conv}}P_{e}/\kappa_{\mathrm{eff}}A_{c}}$, in which *h*_conv_, *P*_e_, and *A*_c_ are the air convective heat transfer coefficient, perimeter, and cross‐section area of the TE‐leaf, respectively; and ∆*T* is the total temperature difference between the heat source and the ambient (∆*T* = ∆*T*_sub_−∆*T*_air_). From Equation (1), the TE‐leaf terminal temperatures were obtained, therefore the temperature difference utilization ratio can be determined as

(2) $$\varphi_{\mathrm{th}}=\left[1-\frac{1}{\cosh\left(\beta L\right)}\right]\frac{h_{b}}{h_{b}+\kappa_{\mathrm{eff}}\beta \tanh\left(\beta L\right)}$$

The open‐circuit voltage *V*_oc_ due to the Seebeck effect is then written as

(3) $$V_{\mathrm{oc}}=\left[1-\frac{1}{\cosh\left(\beta L\right)}\right]\frac{h_{b}\left(S_{p}+S_{n}\right)\Delta T}{h_{b}+\kappa_{\mathrm{eff}}\beta \tanh\left(\beta L\right)}$$

The maximum output power is directly given by

(4) $$P_{\max}=N\frac{V_{\mathrm{oc}}^{2}}{4R_{i}}=N\frac{w{\left(S_{p}+S_{n}\right)}^{2}}{4\left(\frac{1}{\sigma_{p}\delta_{p}}+\frac{1}{\sigma_{n}\delta_{n}}\right)}\cdot \frac{\Delta T^{2}\varphi_{\mathrm{th}}^{2}}{L}$$

where *N* is the number of leaves in a leaf‐TEG, *w* is the width of TE materials, *σ*_p_ and *δ*_p_ are the electric conductivity and thickness of the p‐type material, respectively, *σ*_n_ and *δ*_n_ are those of the n‐type material, respectively, and *S*_p_ and *S*_n_ are the absolute values of the Seebeck coefficient of p‐type material and n‐type material, respectively.

The *P*_max_ curves and *φ*_th_ predicted by Equation (4) are also presented in Figure 2d–f by the solid lines, comparing with measurement results. *P*_max_ is determined by three aspects, i.e., the total temperature difference ∆*T*, the internal electric resistance *R*_i_ and the temperature difference utilization ratio *φ*_th_. Among the three aspects, ∆*T* and *R*_i_, as well as their impacts on the output power, could be determined in a straightforward way in Equation (4). In comparison, *φ*_th_ presents more complicated and nonmonotonically dependences on the leaf dimensions and the air conditions, making itself a key point to the understanding of the response of the output power to various parameters. The relationship between *v*_air_ and *h*_conv_ during tests was given during the theoretical evaluation as Equation (S8) (Supporting Information), and a qualitatively reasonable agreement between the analytical results and the measured data was achieved.

Now we go back to give a comprehensive analysis on *φ*_th_ of the leaf‐TEG. It can be seen from the formula that *φ*_th_ is affected by *h*_b_, *h*_conv_, *κ*_eff_, and the dimensions (mainly length *L*, and thickness *δ*) of the leaves. In the current work for thin film devices, the thermal conductivity and thickness of the TE‐leaf were fixed, thus allowing focus on the remaining factors, specifically *h*_b_, *h*_conv_, and *L*. To perform a control variable analysis, these factors were assumed to be independent on each other. From Equation (2); and Equation (S8) (Supporting Information), we obtained the evolution curves of *φ*_th_ versus TE‐leaf lengths *L* under different *v*_air_ (in Figure 2e). With an increase of *v*_air_, *φ*_th_ experiences a rise at very low air velocity, followed by a slow drop at a higher *v*_air_. At a low *v*_air_, *φ*_th_ is dominated by the TE‐leaf length *L*, and a longer TE‐leaf results in a larger *φ*_th_. While under high *v*_air_, the effect of *L* diminishes and that of *h*_b_ is magnified. All these results were well confirmed by the measured results.

In fact, the influences of *v*_air_ and *L* on *φ*_th_ can be unified into the dimensionless parameter *βL*. At a small *βL*, the second term in the right‐hand side of Equation (2) can be ignored and *φ*_th_ has an approximated form as

(5) $$\varphi_{\mathrm{th}}\approx \frac{{\left(\beta L\right)}^{2}}{2+{\left(\beta L\right)}^{2}}$$

In this situation, *φ*_th_ could be effectively improved by lengthening the leaves. However, at sufficiently large *βL* values, the formula of *φ*_th_ can be simply written as

(6) $$\varphi_{\mathrm{th}}\approx \frac{h_{b}}{h_{b}+\kappa_{\mathrm{eff}}\beta }$$

In this case, one cannot improve *φ*_th_ by utilizing a longer TE‐leaf. Moreover, increasing the length means increasing the internal resistance, thereby reducing the output power.

### 2.4 Sensitivity, Flexibility, and Durability of Leaf‐TEG

**Figure 3:**
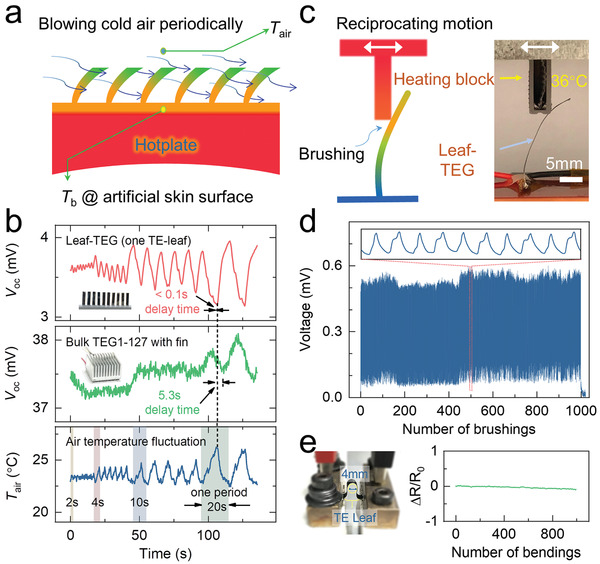
The sensitivity, flexibility, and durability of leaf‐TEG. a) The schematic diagram of the sensitivity to air temperature fluctuation test by blowing cold air periodically. b) Timing diagram of the response characteristic and sensitivity of leaf‐TEG and commercially available rigid TEG module with cooling fin for air temperature fluctuation. The length of different periods is shown by the different widths of the shaded area. c) The schematic diagram of the durability test process, and the heating block is 36 °C. d) Overall and detailed open circuit voltage recording during over 1000 bidirectional brushing on one TE‐leaf. e) Bending tests to confirm the reliability and flexibility over 1000 times (bending radius: 2 mm).

Another advantage of the leaf‐TEG is its sensitive voltage response to air flow fluctuation due to the small heat capacity and the efficient heat transfer through its lateral surface. For a quantitative characterization, we conducted air temperature fluctuations by transiently blowing cold air (≈22 °C) in an air duct system with time periods of 2, 4, 10, and 20 s (**Figure** **3a**). The open‐circuit voltages of 10‐leaf‐TEG (leaves leaf size: 4 × 20 × 0.15 mm^3^) and commercially available rigid TEG module with cooling fins (TEG‐127, 127 Bi_2_Se_0.3_Te_2.7_/Bi_0.5_Sb_1.5_Te_3_ couples, pellet cross‐sectional area of 1.4 × 1.4 mm^2^, pellet height of 1.6 mm) were recorded simultaneously. As shown in Figure 3b; and Figure S10 (Supporting Information), the leaf‐TEG promptly (delay time < 0.1 s) captures the temperature fluctuation (0.2 °C) and generates corresponding voltage fluctuation (70 µV) even under a short period of 0.2 s. In comparison, even under a stronger and longer air temperature fluctuation with a period of 10 s and amplitude up to 2.5 °C, TEG‐127 does not exhibit a notable response under the same air temperature amplitude. A distinguishable voltage fluctuation is observed only as air blowing period up to 20 s, while air temperature amplitude is up to 3.5 °C. It is also noted a hysteresis of 5 s between the temperature stimulate amplitude and voltage response fluctuation for the rigid TEG. If we only compare single p–n pair in both the leaf‐TEG and commercial TEG module, the *V*_oc_ is 80 µV for the leaf‐TEG, while 5 µV for the commercial TEG module. This further reinforces the benefit of the leaf‐TEG for the energy harvesting from the environment with limited temperature difference and air convention.

Furthermore, for an actual application scenario, the flexibility and durability play a crucial role to determine the life time. Figure 3c,d shows a brushing test over 1000 cycles by applying a heating block (36 °C) brushing on the TE‐leaf repeatedly in both directions. The *V*_oc_ and *R*_i_ of the single‐leaf‐TEG was recorded and no circuit disconnection occurred. The bending test of TE‐leaf was also carried out at a radius of 2 mm (Figure S10, Supporting Information). The change of internal resistance is negligible, which is within 2% before and after bending 1000 times as shown in Figure 3e.

We have selectively recorded the output characteristics of the device in the past 13 months in a normal indoor environment (15–25 °C, RH: 50–70%), and there is no obvious deviation in the performance of the device (Figure S11a, Supporting Information). The effects of humidity on the performances of IL‐PEDOT:PSS film and leaf‐TEG are shown in Figure S11b–f (Supporting Information). Consistent with previous reports,^[^ ^32^ ^]^ due to the presence of ions in the film, humidity has a slight influence on the Seebeck coefficient of the IL‐PEDOT:PSS film, but it has no obvious influence on the final power output. It should be noted that the increase in humidity will increase the connection resistance. This is the weakness of using silver paste to connect the circuit, and there is still room for further optimization.

### 2.5 Response and Performance Driven by Human Body Heat

**Figure 4:**
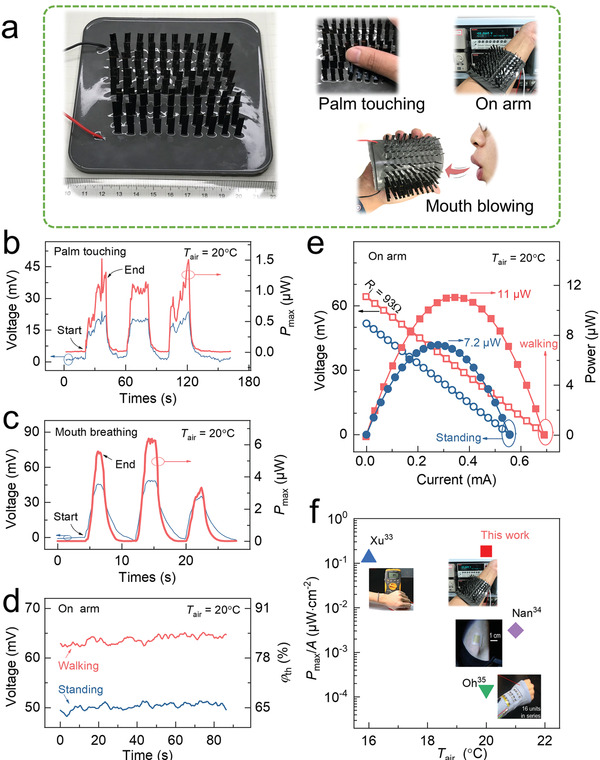
The response and performance measurements of a 100‐leaf‐TEG at room temperature driven by human body heat. a) Proof‐of‐concept of 100‐leaf‐TEG and three actual energy harvesting mode from the human body heat: palm touching, mouth blowing, and worn on the arm. We touched 100‐leaf‐TEG with palm every 20 s for 10 s; and blew it with mouth for 5 s every 10 s. Open‐circuit voltage and the corresponding *P*_max_ of b) the palm touching mode and c) the mouth blowing mode. d) Open‐circuit voltage and corresponding *φ*_th_, and e) power output of the 100‐leaf‐TEG module on an arm while walking and standing situation. The internal resistance of 100‐leaf‐TEG is 93Ω. f) The normalized output performance of leaf‐TEG compared with several literature data points. *A* in f) is the device area including the substrate.

In this section, we assembled a 100‐leaf‐TEG (100 identical TE leaves standing on a substrate of 7.8 × 7.8 cm^2^) for the energy harvesting from the human body heat at room temperature (**Figure** **4a**), including palm touching, mouth blowing and wearing on the arm. In the palm touching mode, 100‐leaf‐TEG was placed on a table with *V*_oc_ monitored in the touch process. Each touching (lasting for 20 s) generates a *V*_oc_ of ≈15 mV and *P*_max_ ≈ 1 µW (Figure 4b). A notable voltage fluctuation is found due to the contact issue between the hot palm and the cool leaf tip. While in the blowing mode, the hot air from the mouth breathing directly blew the 100‐leaf‐TEG on a table. Each blowing (5 s) generated a *V*_oc_ of ≈35 mV and *P*_max_ of ≈4 µW (Figure 4c), which is much higher than that of the touching mode. Both the palm touching and the mouth blowing operation suggest that the as‐fabricated leaf‐TEG is good at harvesting energy from transient heat sources. Furthermore, we also conducted tests while wearing on the arm, which can provide a stable heat source (Figure 4d,e). In a room environment with an ambient temperature of 20 °C, a stable *V*_oc_ of ≈52 mV and an output power of 7.2 µW were obtained when standing still. While walking at a speed about 1 m s^−1^ and swinging the arms, a larger stable *V*_oc_ of ≈64 mV and a higher output power of 11 µW were obtained, corresponding to nearly 0.1 µW for each leaf and a *φ*_th_ of 83%.

## 3 Discussion

The leaf‐TEG takes the advantage of high temperature difference utilization ratio. For an actual application, the power density is still a main concern. For the as‐fabricated 100‐leaf‐TEG, standing on a substrate of 7.8 × 7.8 cm^2^, the power density was about 0.18 µW cm^−2^ when worn on the arm, which is superior to previously reported flexible organic TEGs (see in Figure 4f; and Table S2, Supporting Information),^[^ ^33^, ^34^, ^35^ ^]^ and even some inorganic Bi_2_Te_3_ and Sb_2_Te_3_ generators.^[^ ^10^ ^]^ However, its power density is less than that of the flexible TEG based on bulk Bi_2_Te_3_ elements,^[^ ^7^, ^8^, ^9^ ^]^ because the organic thin film has an intrinsically inferior thermoelectric figure of merit. It is noted that the fill factor of as‐fabricated 100‐leaf‐TEG is only 1%. The packing density could be further increased 5–10 times with extra insulating protection since the fill factor is also an important factor affecting output performance.^[^ ^9^, ^18^, ^36^ ^]^ Furthermore, if the performance of organic thermoelectric materials could be further increased up to the level of the classic Bi_2_Se_0.3_Te_2.7_ and Bi_0.5_Sb_1.5_Te_3_,^[^ ^37^ ^]^ as shown by the prediction results in Figure S12 (Supporting Information), 20 times higher output power would be expected under the same air conditions and element structure dimensions. As an example, if combining both the higher filling factor and improved thermoelectric *ZT*, a predicting value of over 10 µW cm^−2^ is expected in the proposed leaf‐TEG under *v*_air_ of 1.0 m s^−1^, *T*_air_ of 10 °C and *T*_sub_ of 36 °C, which would be very competitive with flexible TEG with bulk TE elements. It is necessary to emphasize the importance of mechanical flexibility of the thermoelectric leaf as compared with the conventional bulk TEG with rigid ceramic plate protection. In the as‐fabricated leaf‐TEG, the hybrid leaf structure with p‐type PEDOT:PSS and n‐type constantan possesses both favorable mechanical flexibility and thermoelectric performance.

## 4 Conclusions

In summary, we proposed a leaf‐TEG, inspired from the grass leaves, by using vertical standing PEDOT:PSS/constantan thin film pairs. The leaf architecture takes the advantage of fully utilizing the temperature difference in the environment with convective heat exchange. The temperature utilization ratio *φ*_th_ is proposed as a performance scale for this new type TEG. In an air duct (substrate: 36 °C, air: 6 °C, air flowing: 1 m s^−1^), the 10‐leaf‐TEG shows a *φ*_th_ of 73% and *P*_max_ of 0.38 µW per leaf with the optimized TE‐leaf length *L* of 10 mm. In the indoor environment with *v*_air_ = 0.5 m s^−1^, *T*_sub_ = 36 °C and *T*_air_ = 25 °C, a *φ*_th_ of 85% is reached with *L* of 20 mm, which is nearly ten times higher than those of commercial TEG modules with cooling fins (9%). We theoretically derivate the analytic formula of *φ*_th_, open circuit voltage *V*_oc_, and output power *P*_max_, which could be a good guideline for further device optimizations. The hybrid p‐type PEDOT:PSS/n‐type constantan thin film leaf is very durable. The change of internal resistance is negligible, which is within 2% before and after bending 1000 times. A proof‐of‐concept wearable 100‐leaf‐TEG (100 leaves, *L* = 10 mm) standing on silicone rubber substrate (7.8 × 7.8 cm^2^) generated 1, 4, and 11 µW in the palm touching, mouth blowing, and arm wearing scenes in an indoor environment, respectively. The proposed leaf‐TEG should be a promising candidate solution for the thermal energy harvesting from environment, particularly for scenarios with limited temperature differences and convection heat transfer conditions.

## 5 Experimental Section

The fabrication and characterization of PEDOT:PSS thin film follow the previous procedure.^[^ ^28^ ^]^ Methods and detailed experimental processes are given in the Supporting Information.

##### Fabrication of the Leaf‐TEG

All of the leaf‐TEG elements are electrically connected in series. Specifically, n‐type and p‐type elements on the top side are connected directly with silver paste, and at the bottom, every n‐type film passes through the insulating layer PI tape to connect to the adjacent p‐type element on the other side. The first n‐type element and the last p‐type element are connected with wires as two independent electrodes. The specific assembly process is shown in Figure 1c.

##### Measurement Setup and Procedure

An electric air ventilator was used for adjusted the air velocity by a programmable DC power supply, and *v*_air_ was monitored in real‐time using a hot wire anemometer (testo 405i). The air temperature was regulated by a temperature‐controllable condenser combined with a refrigerated‐heating circulator (JULABO F32‐MA). A 4 mm thick silicone rubber layer and a 300 µm thick PI film were stuck together as an artificial skin for a similar thermal resistance of the human skin (Figure S4, Supporting Information). The leaf‐TEG was placed on the artificial skin on a PID controlled hotplate (DLAB HP380‐Pro) at 36 °C in the room. The infrared thermal image camera (FLIR E75) was used to measure the temperature distribution on the TE‐leaf. Temperature data for numerical analysis were recorded by NI compact DAQ chassis (cDAQ‐9185), NI‐9214 temperature input module, independent type‐T thermocouples placed in the exact locations (Figure S4, Supporting Information), and LabVIEW software. Here, the temperature difference on TE materials was also calculated by open circuit voltage through the relation of ∆*T*_TE_ = *V*_oc_/(*N∙S*_pn_), where *N* is the number of the TE legs, *S*_pn_ is the Seebeck coefficient of one TE legs. Keithley 2450 SourceMeter was used to record the *I–V* parameter and output power performance. Each measurement data point is an average of 5 min that was recorded after the corresponding *T*_air_ and *V*_air_ for a period of 15 min. A Keithley 2182A Nanometer was used to record the voltage signal of TE device.

##### Statistical Analysis

All experiments were performed at least twice with similar results. For summarizing of TEG performances analyses (Figure 2b–g), data shown are the average values ± standard deviations of 300 independent measurements. Statistical tests were two‐sided if not mentioned otherwise. Statistical analysis was performed using originpro, version 95E.

## Conflict of Interest

The authors declare no conflict of interest.

## Supporting information

Supporting Information

## Data Availability

The data that support the findings of this study are available from the corresponding author upon reasonable request.
