# Supplementary material for: Leaf‐Inspired Flexible Thermoelectric Generators with High Temperature Difference Utilization Ratio and Output Power in Ambient Air
Source: Adv Sci (Weinh). 2021 May 9;8(12):2004947. doi: 10.1002/advs.202004947 (PMC8224459; doi:10.1002/advs.202004947)
Supplement: Supplementary file 1 — Supporting Information [file ADVS-8-2004947-s001.pdf]

© 2021 Wiley-VCH GmbH

ADVANCED SCIENCE
Open Access

Supporting Information

for *Adv. Sci.*, DOI: 10.1002/advs.202004947

Leaf-inspired flexible thermoelectric generators with high temperature difference utilization ratio and output power in ambient air

*Qing Zhou, Kang Zhu, Jun Li, Qikai Li, Biao Deng, Pengxiang Zhang, Qi Wang, Chuanfei Guo, Weichao Wang, and Weishu Liu**

Supporting Information

Leaf-inspired flexible thermoelectric generators with high temperature difference utilization ratio and output power in ambient air

*Qing Zhou, Kang Zhu, Jun Li, Qikai Li, Biao Deng, Pengxiang Zhang, Qi Wang, Chuanfei Guo, Weichao Wang, Weishu Liu**

Supplemental Note S1: Preparation and properties of TE materials

S1.1 Fabrication of PEDOT:PSS flexible films.

Ionic liquid (IL) modified PEDOT:PSS was prepared following the reported procedure.[1] The ionic liquid bis(trifluoromethane) sulfonimide lithium salt (LiTFSI, 99%) was purchased from Aladdin, PEDOT:PSS with PH 1000 grades (the solid content of approximately 1.3 wt.%) was purchased from Clevios, and silver paste was purchased from Ted Pella Inc. All of these chemicals were used as received without further purification. In a typical experiment, LiTFSI (39 mg) was added to the PEDOT:PSS aqueous solution (10 mL). After 15 min of vigorous stirring, the mixture was centrifuged at 5,000 rpm for 1 min to remove the air bubbles, and then poured into a 100 mm × 100 mm disposable PET or PTFE pre-cleaned petri dish. The petri dish was then placed in a vacuum drying oven horizontally for 24 h at 35°C to remove the water. The IL/PEDOT:PSS film was soaked and rinsed in alcohol for 3 times, and then the free-standing IL/PEDOT:PSS film (size: 100 mm × 100 mm) was removed from the petri dish. Then, the IL/PEDOT:PSS film was clamped with cardboard and annealed in a drying oven at 90°C for 4 hours.

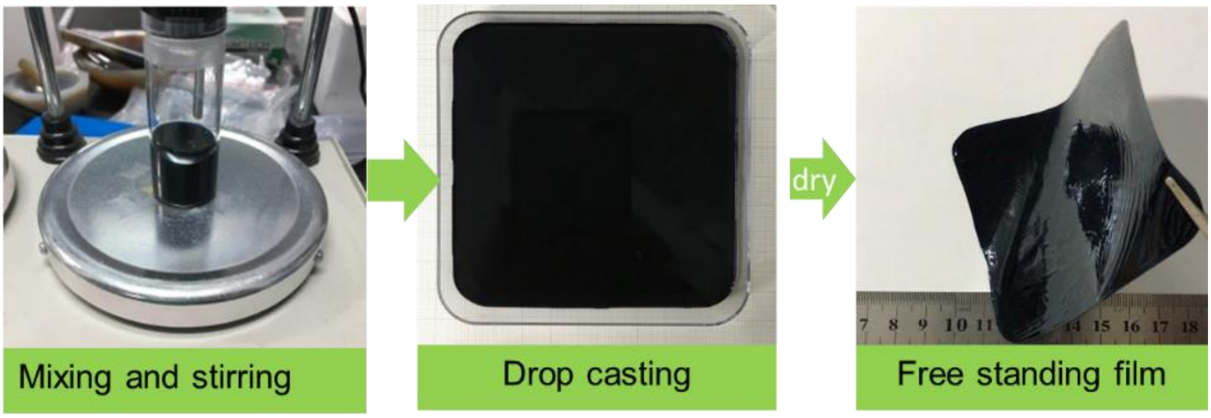

Figure S1 The preparation process of large-area (100 mm × 100 mm) flexible IL/PEDOT:PSS free-standing film. IL=Ionic Liquid, bis(trifluoromethane) sulfonimide lithium salt (LiTFSI). If a larger size mold is used, a larger size film can be prepared. The film preparation method is convenient and practical.

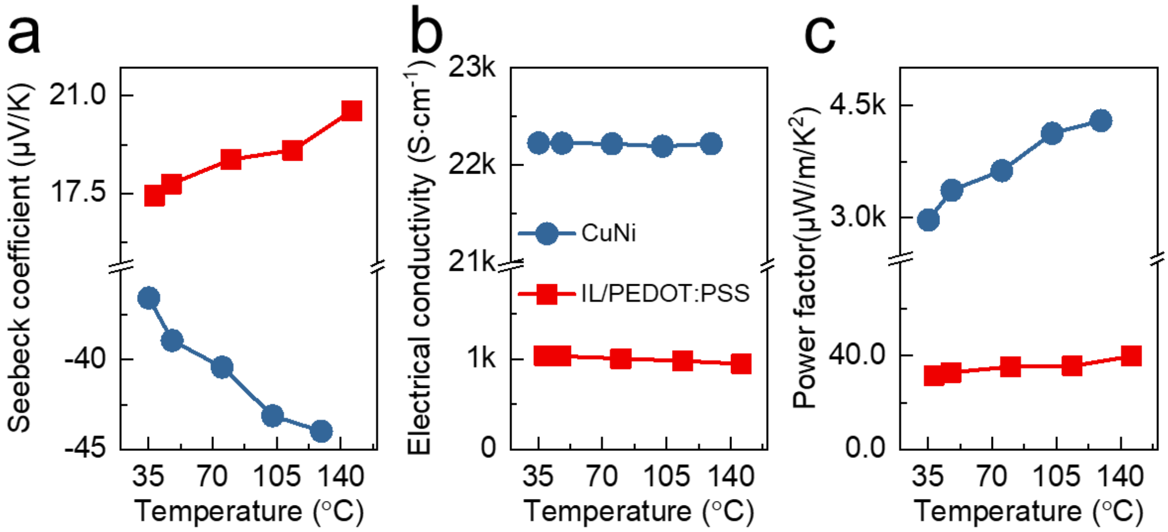

Figure S2 Thermoelectric properties of IL/PEDOT:PSS and constantan thin films. (a) Seebeck coefficients. (b) Electrical conductivities. (c) Power factors. IL=Ionic Liquid, Bis(trifluoromethane) sulfonimide lithium salt (LiTFSI).

S1.2 Characterization of materials.

Scanning electron microscopy (SEM, ZEISS, Merlin) was used to observe the microstructure of the TE films and devices. The in-plane electrical conductivity, Seebeck coefficient, and power factor were measured by CTA-3 (Cryoall Thermoelectric Analysis, China). The dimensions of the sample used for the measurement was about 20 mm × 4 mm

cut from the original free-standing film. Silver paste was coated onto the two ends for a better contact with measurement probes. The thickness and width of the as-prepared thin films were measured by step profiler system (KLA D120) and optical microscope, respectively.

S1.3 Fabrication of the leaf-TEG.

The IL/PEDOT:PSS film (50 μm) was used as the p-type layer of the leaf TEG, while the commercial available constantan foil ($Cu_{55}Ni_{45}$, thickness: 5 μm, Goodfellow) was used as the n-type TE film. For a single thermoelectric leaf, it was stacked together with a PI film in an order of p-i-n, while the tip of the leaf is electrical joint by the silver paste (Figure 1c). The specific assembly process is shown in the figure below and also given in Figure S3.

The width of the leaf is 4 mm, while the length is 5 mm, 10 mm, 15 mm and 20 mm. For a leaf-TEG, the leaves were place on the thermally conductive silicone rubber substrate (SINWE905, thickness: 3 mm) with a thin PDMS layer as heat insulate (PDMS: polydimethylsiloxane, thickness: 0.5 mm, thermal conductivity: ~0.15 $W \cdot m^{-1} \cdot K^{-1}$). For the leaf-TEG with 10 leaves, it is expressed as 10-leaf-TEG, and the substrate size of which is 7.8 mm×0.8 mm. While for the leaf-TEG with 100 leaves, expressed as 100-leaf-TEG, the substrate size is 7.8 mm×7.8 mm.

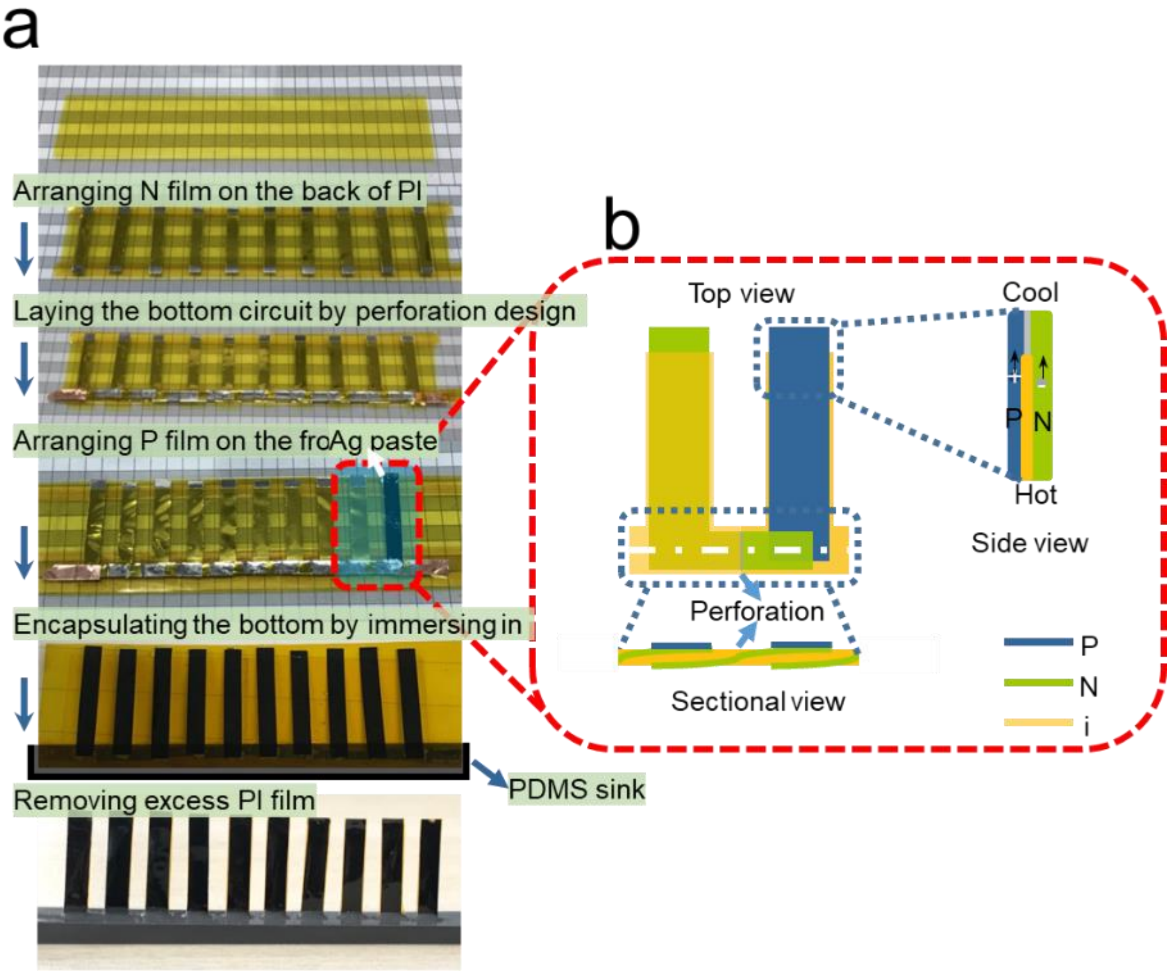

Figure S3 Design and fabrication of the 10-leaf-TEGs. (a) Schematic illustration of the design, fabrication process, and key characteristics, including back-to-back PN TE legs and optical images of 10-leaf-TEGs. (b) The Three View of leaf-TEGs for clearly showing the circuit connection.

S1.4 Installation of the lab-made air duct system.

For achieving quantitative measurements, an air duct system was designed (Figure 2a). An electric air ventilator was used for adjusted the air velocity by a programmable DC power supply, and v_{air} was monitored in real-time using a hot wire anemometer (testo 405i). The air temperature was regulated by a temperature-controllable condenser combined with a refrigerated-heating circulator (JULABO F32-MA). A 4-mm thick silicone rubber layer and a 300-μm thick PI film were stuck together as an artificial skin for a similar thermal resistance

of the human skin (Figure S4). The leaf-TEG was placed on the artificial skin on a PID controlled hotplate (DLAB HP380-Pro) at 36°C in the room.

S1.5 Measurements of temperature difference utilization ratio and output power performance.

The infrared thermal image camera (FLIR E75) with a thermal sensitivity of 0.03°C and its supporting software (FLIR Tools) was used to directly measure the temperature distribution on the TE-leaf. Temperature data for numerical analysis were measured and recorded by NI compact DAQ chassis (cDAQ-9185), NI-9214 temperature input module, independent type-T thermocouples placed in the exact locations (Figure S4), and LabVIEW software. Here, the temperature difference on TE materials was also calculated by open circuit voltage through the relation of $\Delta T_{TE}=V_{oc}/(N\cdot S_{pn})$, where N is the number of the TE legs, S_{pn} is the Seebeck coefficient of one TE legs. Keithley 2450 SourceMeter was used to record the *I-V* parameter and output power performance of the leaf-TEG under current scanning mode for achieving the equivalent effect of switching the load resistance. Each measurement data point is an average of 5 min (300 measuring points) that was recorded after the corresponding T_{air} and V_{air} for a period of 15 min. A Keithley 2182A Nanometer was used to record the voltage signal of TE device.

S1.6 Tests of flexibility and durability.

A bending test was conducted on a lab-made apparatus with precise controlled translation stage. The internal resistance of the TE-leaf was measured by Keithley 2182A. For durability testing, a heating block (36°C), which enclosed in the aluminum shell, was placed on a motorized linear stage (Zolix PSA100-11-X) and moved backward and forward brushing the TE-leaf (4 mm × 30 mm) bi-directionally. The overlap distance between the top of the device and the bottom of the heating block is 5 mm (1/4 of TE-leaf length). All of the

measurements were completed in an indoor environment with a temperature of approximately 25°C.

Supplemental Note S2: Measurements in air ambience

We set up an air duct system for the power generation measurement with controllable air temperature and air flowing speed. A PID controlled hotplate is used for providing heat continuously as human body core. 4-mm thick silicone rubber, 300 μm PI film, and 2 mm polyacryamide hydrogel are closely contacted layer by layer, so that the heat resistance of which would be similar to the conditions of a human skin.

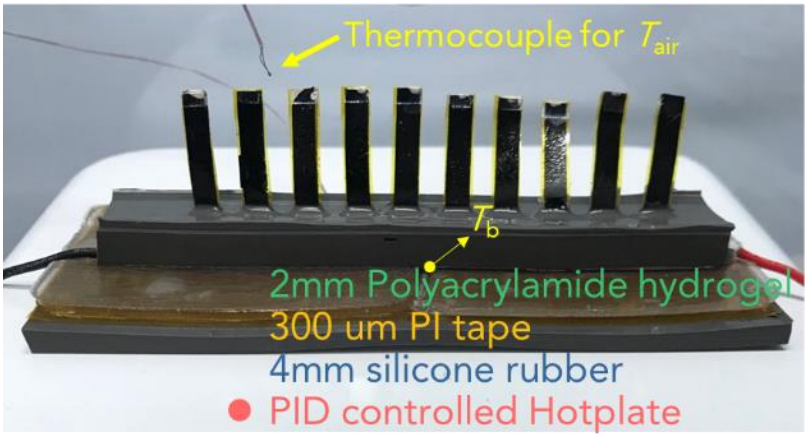

Figure S4 The artificial skin and its placement in the air duct system for the power generation measurement. The location measuring T_{sub} and T_{air} were pointed out.

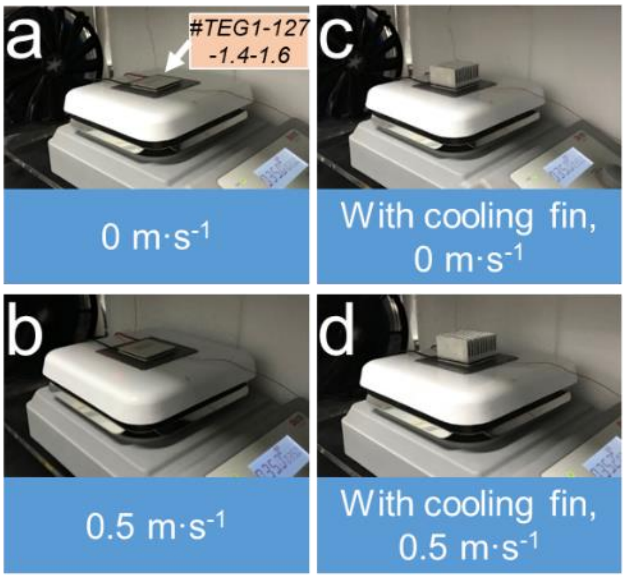

Figure S5 Temperature utilization ratio φ_{th} measurement of commercial thermoelectric generator module TEG1-127-1.4-1.6 different conditions. (a) and (b) without and with cooling fin at static state. (c) and (d) without and with cooling fin under an air flow speed of 0.5 m·s^{-1}. The measured results of φ_{th} are shown in Table S1.

Table S1 φ_{th} of commercial module TEG1-127 in Figure S5 at room temperature.

T_{air} [°C]	T_{h} [°C]	V_{air} [m·s^{-1}]	**Cooling fin**	V_{oc} [mV]	φ_{th} [%]
22	35	0	Without	16	2.4
		0.5		32	4.8
		0	With	55	8.0
		0.5		64	9.3

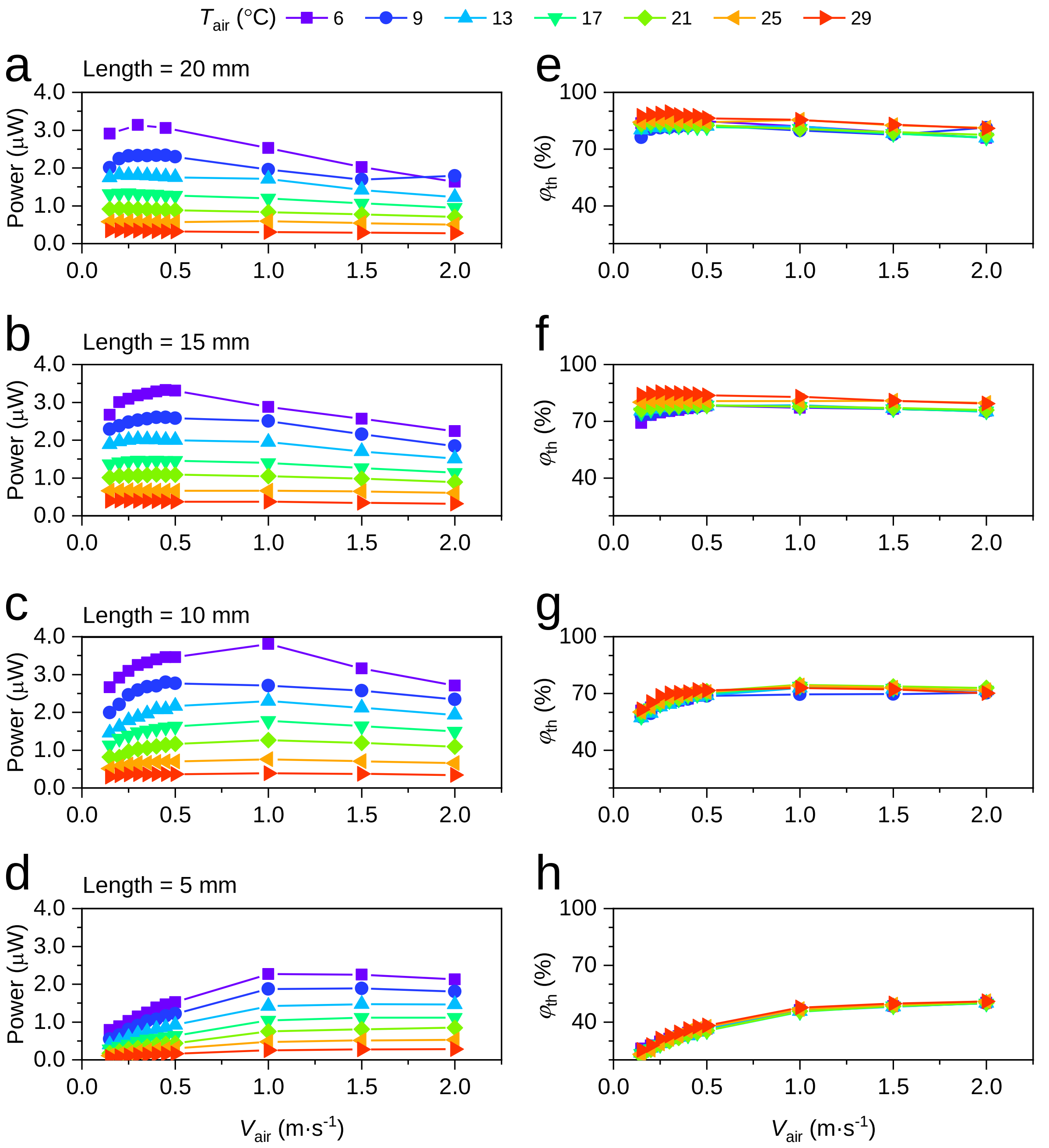

Figure S6 The effects of the air temperature from ~6°C to ~29°C, air velocity from 0.15 m·s^{-1} to 1.0 m·s^{-1} and leaf dimensions on P_{max} and φ_{th} of 10-leaf-TEG. (a)-(d) Detailed measurement results of the maximum output power P_{max}. (e)-(h) Detailed measurement results of temperature utilization ratio φ_{th}. Four different leaf lengths are 20, 15, 10 and 5 mm, respectively.

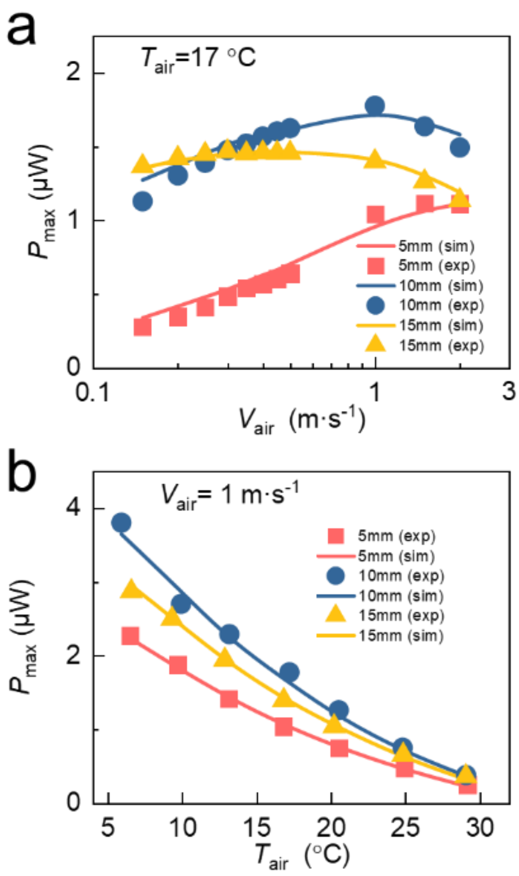

Figure S7 Effects of V_{air} (a) and T_{air} (b) on the output power performance of leaf-TEG.

The influence of V_{air} on P_{max} is more complicated than that of T_{air} on P_{max}, since the former is determined by the form of φ_{th}^2/R_i and substrate cooling.

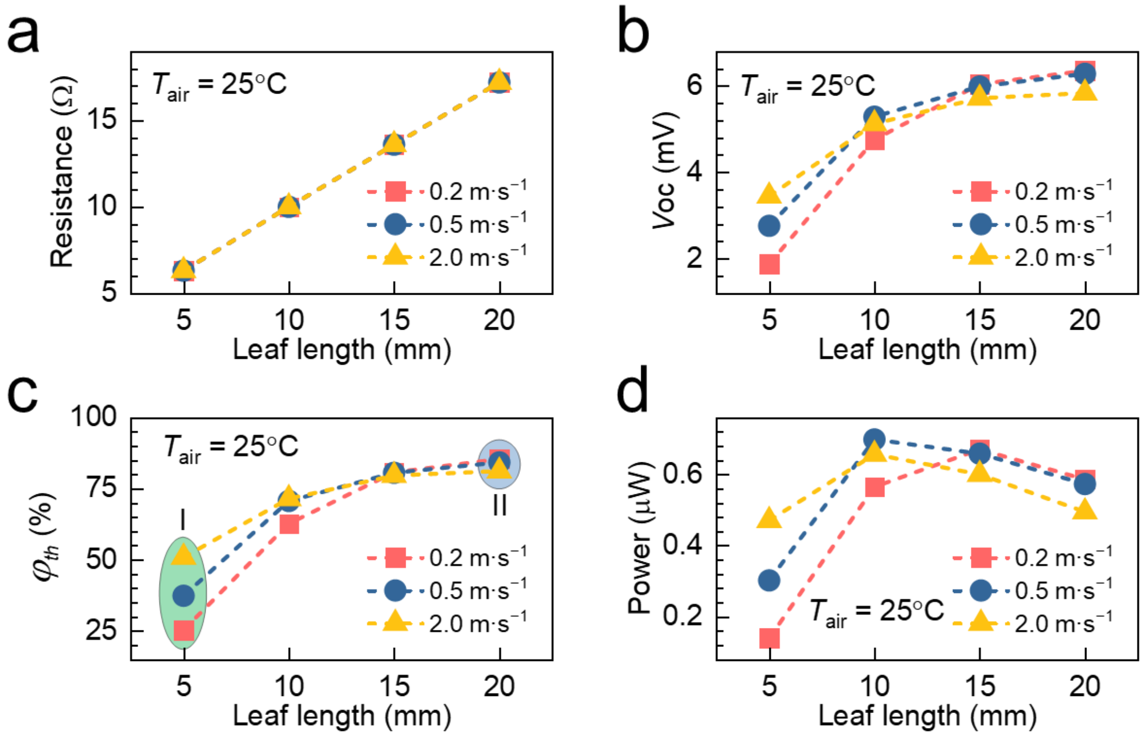

Figure S8 Effects of V_{air} and T_{air} on the output power performance of leaf-TEG with different leaf lengths under T_{air} of 25°C. (a) Internal resistance R_i, (b) Open-circuit voltage V_{oc}, (c) temperature difference utilization ratio φ_{th} and (d) maximum output power P_{max} of leaf-TEG with different length under different air velocity conditions (0.2, 0.5, and 2.0 m·s^{-1}).

Supplemental Note S3: Theoretical derivation of temperature utilization ratio φth and output power P with a heat transfer model.

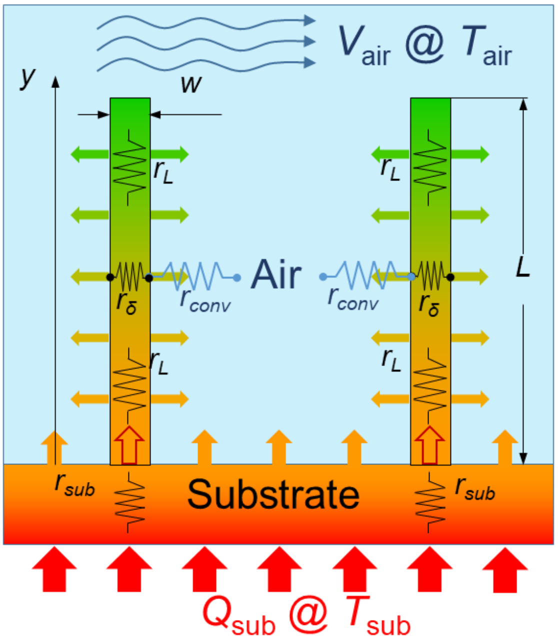

Figure S9 One-dimensional fin heat transfer model for leaf-TEG.

We introduce the one-dimensional heat transfer theory module (Figure S9) to investigate the establishment of temperature difference within a leaf-type TEG standing in a static or flowing air environment. The leaf absorbs heat from the substrate, and continuously releases heat at its lateral surface, resulting in a descending temperature profile along the vertical direction. Firstly, the ratio between the longitudinal TE-leaf thermal resistance (r_δ) and the convective resistance of the surrounding air (r_{conv}) is checked, to confirm the rationality of a one-dimensional heat transfer analysis on the TEG discussed here,

$$\frac{r_\delta}{r_{conv}} = \frac{h_{conv}\delta}{\kappa_{eff}} \#(S1)$$

where h_{conv} is the convective heat transfer coefficient, δ is the TE-leaf thickness, and κ_{eff} is the effective thermal conductivity of the TE-leaf, which could be calculated by a weighted average of the thermal conductivities of the three components, *i.e.*, $\kappa_{eff} = \frac{\kappa_P A_P + \kappa_N A_N + \kappa_I A_I}{A_c}$, where κ_P and A_P, κ_N and A_N, κ_I and A_I are the thermal conductivities and cross-section areas of the P type material, the N type material and the intermediate adhesive layer, respectively, and A_c is the total cross-section area of the TE leaf. In the current situation, the convective heat transfer coefficient falls into a range of 10^0~10^2 $W \cdot m^{-2} \cdot K^{-1}$, the TE-leaf thickness is around 0.1 mm and the effective thermal conductivity is on the order of 1 $W \cdot m^{-1} \cdot K^{-1}$, leading to a longitudinal thermal resistance ratio lower than 0.01, which is well suitable for a longitudinally-uniform temperature approximation, thus a one-dimensional heat transfer analysis is performed in the vertical direction.

The vertical temperature distribution within the TE-leaf is a result of the competition among the thermal resistances of the substrate r_{sub}, the TE-leaf r_L (in the vertical direction) and the air convection r_{conv} (in the longitudinal direction). By solving the following steady-state governing equation and boundary conditions, the temperature distribution along the length of the TE-leaf is obtained,

$$\begin{cases} \kappa_{eff}\dfrac{\mathrm{d}^2T}{\mathrm{d}y^2}+\dot{\Phi}=0 \quad (a) \\ -\kappa_{eff}\dfrac{\mathrm{d}T}{\mathrm{d}y}=h_b(T_{sub}-T),\ \ y=0 \quad (b) \\ \dfrac{\mathrm{d}T}{\mathrm{d}y}=0,\ \ y=L \quad (c) \end{cases} \#(\mathrm{S2})$$

where L is the length of the TE-leaf, T_{sub} is the heat source (artificial skin or human skin) temperature, and h_b is the equivalent heat transfer coefficient between the TE-leaf root and the heat source. The heat transfer between the TE-leaf surface and the surrounding air is converted into a volumetric heat source term in the governing equation as $\dot{\Phi}=\frac{h_{conv}P_e}{A_c}(T_{air}-T)$, in which T_{air} is the ambient temperature, P_e is the perimeter and A_c is the cross-section area of the TE-leaf. As a primary model, the impact of electric current (including the Peltier effect and the Joule effect) on the temperature distribution is overlooked to offer a concise physical picture, which is well acceptable for TEGs with relatively low Seebeck coefficients and limited temperature differences. The solution of Equation S2 is written as

$$T=\frac{h_b}{h_b+\kappa_{eff}\beta\tanh(\beta L)}\frac{\cosh(\beta y-\beta L)}{\cosh(\beta L)}\Delta T+T_{air}\#(\mathrm{S3})$$

where $\Delta T=T_{sub}-T_{air}$ represents the total temperature difference between the heat source and sink, and $\beta=\sqrt{\frac{h_{conv}P_e}{\kappa_{eff}A_c}}$. From Equation S3, the TE-leaf terminal temperatures and the effective temperature difference are obtained,

$$\begin{cases} T_0=\dfrac{h_b\Delta T}{h_b+\kappa_{eff}\beta\tanh(\beta L)}+T_{air} \quad (a) \\ T_L=\dfrac{1}{\cosh(\beta L)}\dfrac{h_b\Delta T}{h_b+\kappa_{eff}\beta\tanh(\beta L)}+T_{air} \quad (b) \\ \Delta T_{eff}=\left[1-\dfrac{1}{\cosh(\beta L)}\right]\dfrac{h_b\Delta T}{h_b+\kappa_{eff}\beta\tanh(\beta L)} \quad (c) \end{cases} \#(\mathrm{S4})$$

The temperature utilization ratio φ_{th} is defined as the ratio of the effective temperature difference (the temperature dropped across the TE leg, ΔT_{eff}) to the total temperature difference between the heat source and sink,

$$\varphi_{th} = \frac{\Delta T_{eff}}{\Delta T} = \left[1 - \frac{1}{\cosh(\beta L)}\right] \frac{h_b}{h_b + \kappa_{eff}\beta\tanh(\beta L)} \#(S5)$$

The open-circuit voltage due to the Seebeck effect is then written as

$$V_{oc} = S\Delta T\varphi_{th} = \left[1 - \frac{1}{\cosh(\beta L)}\right] \frac{h_b S\Delta T}{h_b + \kappa_{eff}\beta\tanh(\beta L)} \#(S6)$$

The maximum output power is directly given by

$$P_{max} = N\frac{V_{oc}^2}{4R_i} = \frac{NS^2}{4\left(\frac{1}{\sigma_P A_P} + \frac{1}{\sigma_n A_n}\right)} \frac{{\varphi_{th}}^2 \Delta T^2}{L}$$

$$= \frac{N}{4L\left(\frac{1}{\sigma_p A_p} + \frac{1}{\sigma_n A_n}\right)} \left[1 - \frac{1}{\cosh(\beta L)}\right]^2 \left[\frac{h_b S\Delta T}{h_b + \kappa_{eff}\beta\tanh(\beta L)}\right]^2 \#(S7)$$

where N is the number of leaves in a leaf-TEG, σ_p and A_p are the electric conductivity and cross-section area of the p-type material, and σ_n and A_n are those of the n-type material, respectively; S is the sum of the absolute values of the Seebeck coefficient of p-type materials and n-type materials, *i.e.*, $S = S_p + S_n$.

To utilize the above theoretical formulae for the performance evaluation of the leaf-TEGs, the heat transfer coefficients, *i.e.*, h_b and h_{conv} need to be determined at first. The equivalent heat transfer coefficient h_b at the root of the leaves is affected by the contact quality as well as the thermal conductivity of the substrate. Once a sample is fabricated, h_b will be on a stable level and is easy to be determined by the measured temperature profiles. In this work, h_b was assessed to be about 1,550 W·m^{-2}·K^{-1}. It is difficult to determine the precise value of h_{conv} during tests, while it is expected to increase with the air velocity and decrease with the TE-leaf width in the current arrangement. We can suppose a simple but rational correlation in the following form to perform a qualitative analysis,

$$h_{conv} = C_0\left(\frac{V_{air}}{w}\right)^n \#(S8)$$

where C_0 and n are constants, and we value them as 0.45 and 0.5 respectively to match a velocity range of 0.1~2 m·s^{-1} with a h_{conv} range of 1.5~10 W·m^{-2}·K^{-1} under an TE-leaf width of 4 mm.

Supplemental Note S4: Sensitivity and environmental reliability test

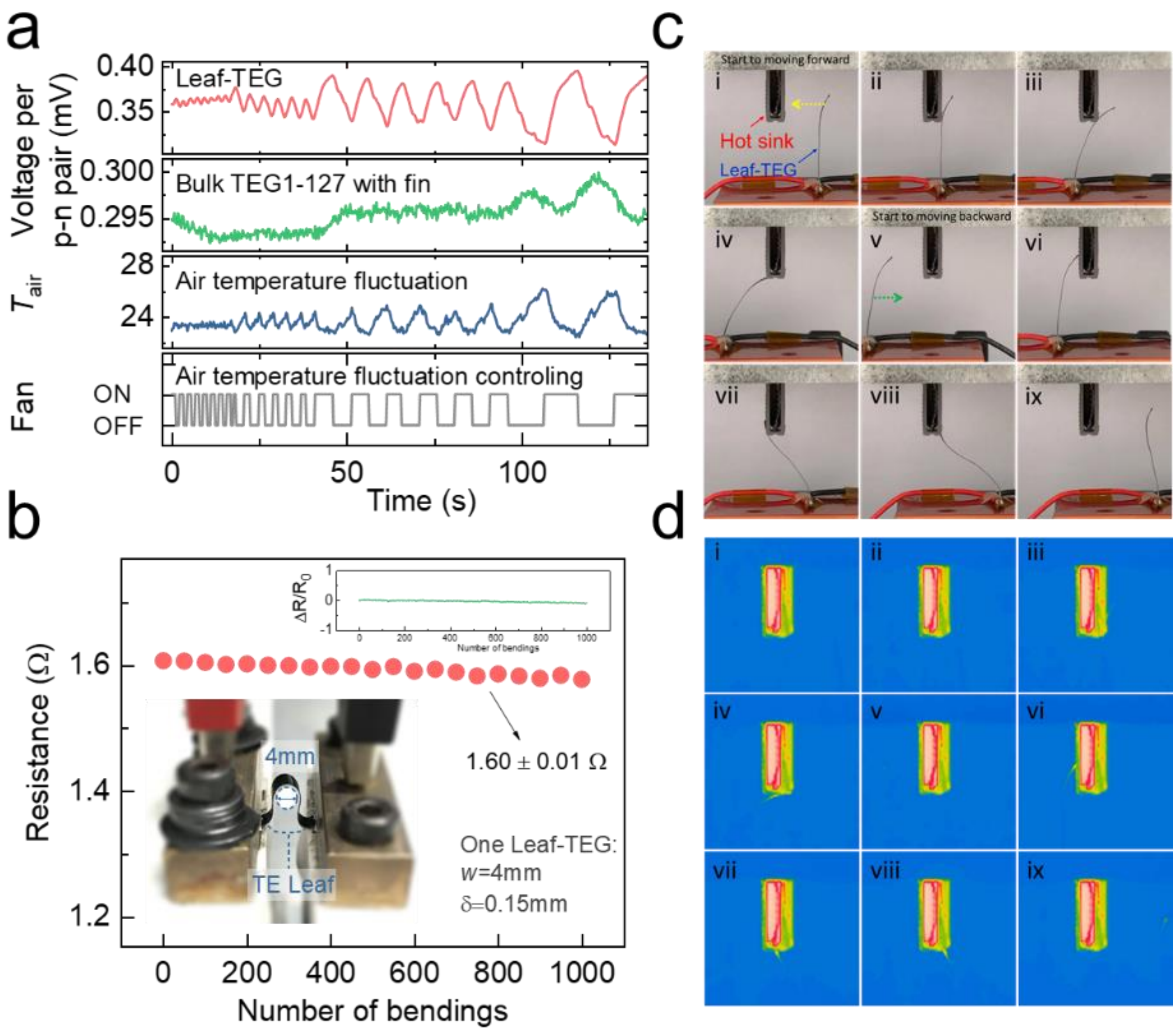

Figure S10 Sensitivity and bending test for leaf-TEG. (a) Timing diagram of the response characteristic and sensitivity of leaf-TEG and commercially available rigid TEG module with cooling fin for air temperature fluctuation. The length of different periods was controlled by shut on or off electronic fan periodically. (b) TE-Leaf bending test. The optical photos (c) and infrared images (d) of leaf-TEG brushing tests at different stages.

We selectively recorded the output characteristics of the device in the past 13 months in a normal indoor environment (15°C -25°C, RH: 40%-70%) as aging test (Figure S11a), and there is no obvious deviation in the performance of the device. The effects of humidity on the performances of IL-PEDOT:PSS film and leaf-TEG were investigated shown in Figure S11b-f. After the film absorbs water, ions can participate in thermoelectric transport to generate additional electric potential.[2] Seebeck coefficients of both film (Figure S11b) and TEG (Figure S11c) show this effect. It should be noted that the increase in humidity will increase the connection resistance (Figure S11d). This is the weakness of silver paste using to connect

the circuit, and there is still room for further optimization in the future. However, the fluctuation of the output performances (V_{oc} in Figure S11e and P_{max} in Figure S11f) were not directly related to the humidity change. This is due to the device is also affected by the temperature of the hot and cold ends, the contact of the hot end, and the air convection, which are greater than the influence of humidity.

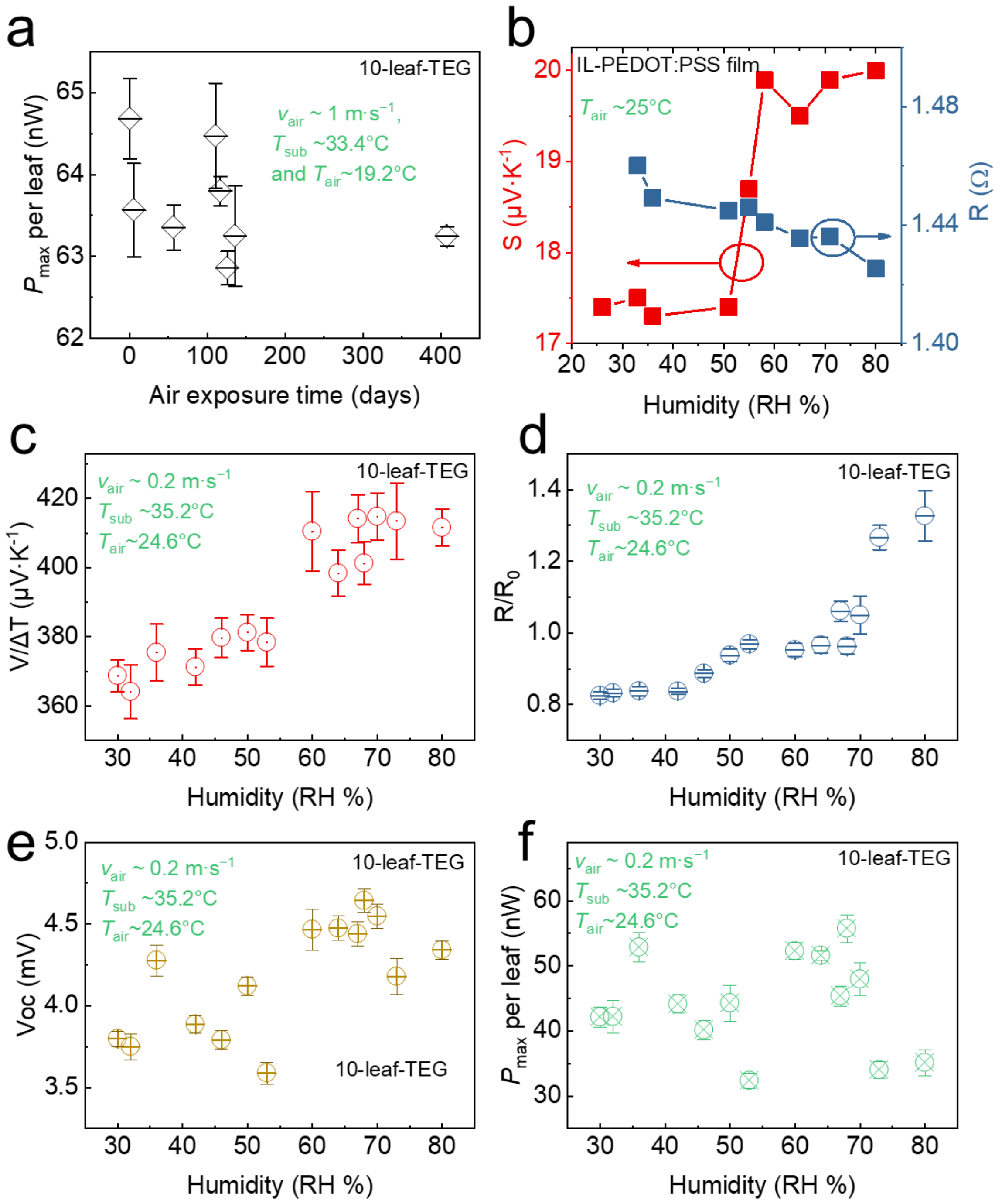

Figure S11 Environmental reliability test of leaf-TEGs. (a) Maximum output power of 10-leaf-TEGs change according with the air exposure time. Keeping indoor (15°C -25°C, RH: 40%-70%) (b) The effects of humidity on the performances of IL-PEDOT:PSS film and (c) to (f) on the leaf-TEGs. (c) Open circuit voltage per unit temperature difference under different

humidity, which has the same changing trend as Seebeck coefficient of IL/PEDOT:PSS film in (b). (d) Resistance, (e) open-circuit voltage and (f) maximum output power per leaf changing with humidity.

Supplemental Note S5: The output power performance comparison and discussion.

Table S2 The output performance and the corresponding φ_{th} of leaf-TEG compared with several literature data points.

Conformation	ΔT [°C]	V_{oc} [mV]	S_P+S_N [μV·K^{-1}]	P-N couples	Area [m^2]	φ_{th} [%]	Power density [μW·m^{-2}]	Power density a) [μW·m^{-2}·K^{-2}]	Ref.
**Glass fabric**	11.9	2.9	239	11	5×10^{-4}	5.6	6000	15	[3]
**Spring**	18	8.9	266	7	5×10^{-4}	27	1320	4.07	[4]
**Spring**	19	51.3	130	64	6.4×10^{-5}	33	31.3	0.087	[5]
**Sheet**	3	2.4	89	16	5×10^{-3}	56	1.5	0.16	[6]
**Leaf-TEG**	14	64	55	100	6×10^{-3}	83	1841	9.4	This work b)
**Leaf-TEG**	30.6	12.3	55	10	6×10^{-4}	73	6342	6.8	This work c)

a) Counting the area of the substrate; b) Usual wearing scene (walking, and T_{air}=29°C); c) Under cold scene (T_{air}=6°C and V_{air}=1 m·s^{-1})

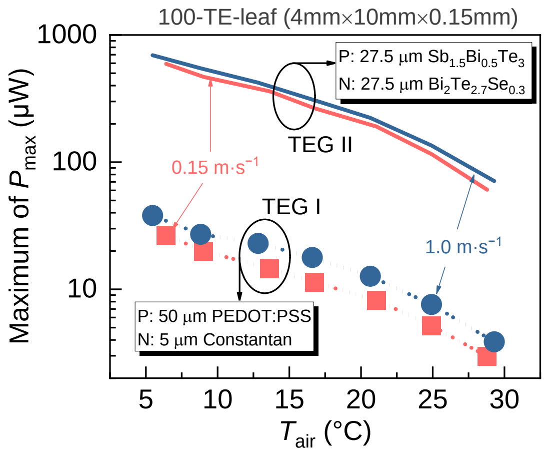

Figure S12 The output power performance comparison of two leaf-TEGs. consisting of two different thermoelectric performances materials respectively. Both two leaf-TEGs were consisted with 10 TE leaves with the same structure size (4 mm × 10 mm × 0.15 mm), and working under the same conditions (T_{air}: 5.5°C - 29°C, V_{air}:0. 5 m·s^{-1} or 1.0 m·s^{-1}). When calculating TEG II, the thickness of the PI tape layer was set as 95 μm, the total thickness P and N TE films was set as 55 μm to ensure the same thickness as TEG I.

References

[1] Q.K. Li, M.J. Deng, S.M. Zhang, D.K. Zhao, Q.L. Jiang, C.F. Guo, Q. Zhou, W.S. Liu, *J. Mater. Chem. C* **2019**, 7, 4374.

[2] H. Wang, U. Ail, R. Gabrielsson, M. Berggren, X. Crispin, *Adv. Energy Mater.* **2015**, 5, 1500044.

[3] S.J. Kim, J.H. We, B.J. Cho, *Energy Environ. Sci.* **2014**, 7, 1959.

[4] X.J. Xu, Y. Zuo, S. Cai, X. Tao, Z.M. Zhang, X.F. Zhou, S. He, X.S. Fang, H.S. Peng, *J. Mater. Chem. C* **2018**, 6,4866.

[5] K.W. Nan, S.D. Kang, K. Li, K.J. Yu, F. Zhu, J.T. Wang, A.C. Dunn, C.Q. Zhou, Z.Q. Xie, M.T. Agne, H.L. Wang, H.W. Luan, Y.H. Zhang, Y.G. Huang, G.J. Snyder, J.A. Rogers, *Sci. Adv.* **2018**, 4, eaau5849.

[6] J.Y. Oh, J.H. Lee, S.W. Han, S.S. Chae, E.J. Bae, Y.H. Kang, W.J. Choi, S.Y. Cho, J.O. Lee, H.K. Baik, T.I. Lee, *Energy Environ. Sci.* **2016**, 9, 1696.
